# Abnormalities in Cutaneous Microcirculation in Patients with Alzheimer’s Disease, Mild Cognitive Impairment, and Chronic Insomnia Disorder

**DOI:** 10.3390/jcm10245718

**Published:** 2021-12-07

**Authors:** Sebastian Yu, Chung-Yao Hsu, Hung-Yi Chuang, Chen-Cheng Yang, Chiou-Lian Lai, Hsin-Su Yu

**Affiliations:** 1 Department of Dermatology, Faculty of Medicine, College of Medicine, Kaohsiung Medical University, Kaohsiung 807378, Taiwan; epor324@gmail.com; 2 Department of Dermatology, Kaohsiung Medical University Hospital, Kaohsiung 807377, Taiwan; 3 Graduate Institute of Clinical Medicine, College of Medicine, Kaohsiung Medical University, Kaohsiung 807378, Taiwan; 4 Neuroscience Research Center, Kaohsiung Medical University, Kaohsiung 807378, Taiwan; 5 Department of Neurology, Kaohsiung Medical University Hospital, Kaohsiung Medical University, Kaohsiung 807378, Taiwan; cyhsu61@gmail.com; 6 Department of Public Health and Environmental and Occupational Medicine, School of Medicine, College of Medicine, Kaohsiung Medical University, Kaohsiung 807378, Taiwan; ericch@kmu.edu.tw; 7 Department of Occupational Medicine, Kaohsiung Municipal Siaogang Hospital, Kaohsiung Medical University, Kaohsiung 807378, Taiwan; abcmacoto@gmail.com; 8 Graduate Institute of Medicine, College of Medicine, Kaohsiung Medical University, Kaohsiung 807378, Taiwan

**Keywords:** autonomic, laser Doppler flowmetry, microangiopathy, parasympathetic, perfusion, sympathetic

## Abstract

Impaired sympathetic response is frequently observed in neurodegenerative diseases, such as Alzheimer’s disease (AD). On the other hand, chronic insomnia disorder (CID) is also often accompanied by activation of sympathetic nerves. Considering that cutaneous microcirculation reflects sympathetic tone, we hypothesized that baseline cutaneous microcirculation in fingers, as detected by laser Doppler flowmetry (LDF), differs among patients with mild cognitive impairment (MCI), AD, and CID. As light therapy is one of the adjunctive treatments for AD and CID, we designed a randomized controlled cross-over trial of light therapy through eyes for 12 weeks with red light as treatment and green light as control limb, and examined if light therapy has an impact on cutaneous microcirculation. Before light therapy, patients with AD had significantly lower baseline cutaneous perfusion than those with CID in left and right first to fourth fingers. After red light therapy, however, cutaneous perfusion of fingers in CID patients significantly decreased (right fingers, before vs. after = 227.25 ± 62.00 vs. 162.00 ± 49.34, *p* = 0.007; left fingers, before vs. after = 228.99 ± 58.80 vs. 177.41 ± 59.41, *p* = 0.003) while cutaneous perfusion of fingers in CID patients did not significantly change after green light therapy. Light therapy with red light also significantly increased cutaneous finger perfusion in patients with AD (right fingers, before vs. after = 130.13 ± 49.82 vs. 172.38 ± 38.32, *p* = 0.043). Our results suggest that cutaneous perfusion is a useful tool to detect sympathetic dysfunction in patients with CID and AD, and that light therapy with red light is a potential therapeutic intervention to reverse impaired sympathetic function in patients with CID and patients with AD.

## 1. Introduction

Laser Doppler flowmetry (LDF) is a non-invasive method to measure local microvascular blood flow in tissues [1,2]. LDF has been used in scientific research to measure cutaneous microvascular damage in certain diseases, such as systemic sclerosis, diabetes mellitus, and hypertension [2,3,4,5]. In these circumstances, cutaneous microvasculopathy is a known potential complication of these diseases, and LDF is used to detect the occurrence or extent of microvasculopathy. LDF could also be adopted as a tool to measure microcirculatory change after interventions [6,7,8,9,10]. For example, a study showed finger microcirculation, as detected by LDF, increases following 3 days of iloprost infusion but vanishes within 7 days in patients with systemic sclerosis, suggesting a longer lasting vasodilatory regimen remains to be established [10]. LDF has also been proposed as an indicator for sympathetic nervous system function [11,12,13,14,15]. In clinical scenarios, LDF and other tools have been used to measure microcirculation of certain diseases with abnormal neural/sympathetic modulation, such as segmental vitiligo [16,17,18] and port-wine stain [19,20], to diagnose these diseases or predict their response to treatment. Specifically, we identified that evaluation of cutaneous microcirculation with and without prior visible light irradiation on cold-stressed segmental vitiligo, a subtype of vitiligo with neural abnormality, may serve as a treatment response predictor. These findings support the concept that normalization of sympathetic dysfunction may account for the efficacy of visible light in treating segmental vitiligo [16]. However, the current and developing clinical applications of LDF are mainly for dermatologic diseases with neural etiology. As is well known, both the skin and the central nervous system derive from the ectoderm of an embryo, and various cutaneous and neuropsychiatric diseases are associated with possible close genetic loci carrying susceptibility genes and/or the brain-skin communications during development [21,22,23,24]. Considering sympathetic nerve activity is an integral part of the central nervous systems, we further asked if central nervous disorders would be accompanied by abnormal cutaneous microcirculation, and if LDF could detect the abnormal cutaneous microcirculation. Specifically, chronic insomnia disorder (CID) and Alzheimer’s diseases (AD) are known to be accompanied by activated and impaired sympathetic response, respectively [25,26,27]. Furthermore, light therapy is one of the adjunctive treatments for AD and CID [28,29,30,31]. In the present study, we investigated baseline cutaneous microcirculation in patients with CID, AD, and mild cognitive impairment (MCI), due to AD and examined change in cutaneous microcirculation after light therapy.

## 2. Materials and Methods

### 2.1. Patients and Study Design

**Figure 1:**
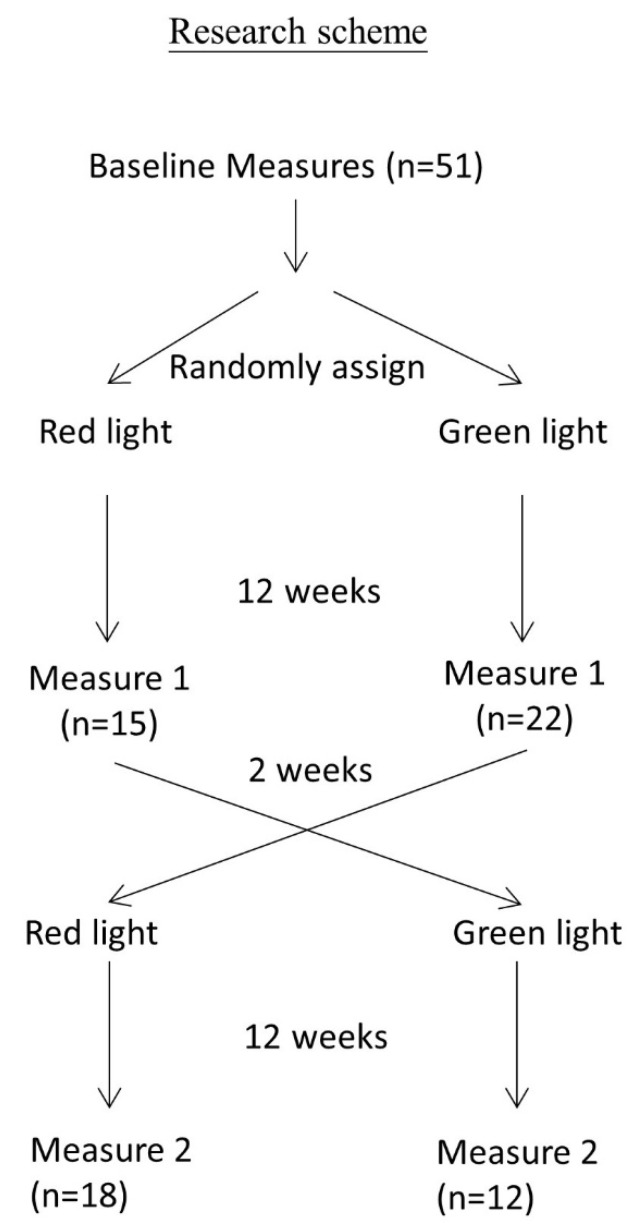
Algorithm of the study design. Cutaneous perfusion of each finger was measured before light therapy. Subjects were randomly allocated to receive green light as treatment or red light as control limb for 12 weeks, and cutaneous perfusion of each finger was measured. After wash-out for 2 weeks, subjects were crossed over to receive green light therapy or red light control, and cutaneous perfusion of each finger was measured again.

Patients who met diagnostic criteria of amnestic mild cognitive impairment (aMCI) or AD were recruited, and patients with CID were used as control subjects. AD and MCI were diagnosed according to the criteria proposed by National Institute on Aging-Alzheimer’s Association Workgroups in 2011 [32]. CID was diagnosed according to the criteria of the third edition of the International Classification of Sleep Disorders (ICSD-3) [33]. Only patients aged between 55–80 years, had an education level of elementary school or above, and with dextromanuality were included in the study. Exclusion criteria were sleep apnea; regular hypnotics or psychostimulants use; photodermatosis; major neurological diseases including stroke, epilepsy, Parkinson’s disease, or head injury; major psychiatric diseases including psychosis, drug addiction, personality disorder, or poor controlled neurotic disorder; major ophthalmologic diseases including retinopathy, macular degeneration, or severe cataract; and major illnesses including cancer, poor controlled diabetes mellitus, poor controlled hypertension, thyroid disease, rheumatic disease, congestive heart failure, chronic obstructive pulmonary disease, liver cirrhosis, or chronic kidney disease stage 4 or 5. As light therapy is one of the adjunctive treatments for AD and CID, we further examined if randomized controlled cross-over trial of light therapy through the eyes for 12 weeks with red light at the wavelength of 660–680 nm as the treatment arm, and green light at the wavelength of 520–535 nm as the control arm had an impact on cutaneous microcirculation. Red light or green light emitted from light-emitting diode (LED) was used as the light source. The LED device was customized by Taiwan Instrument Research Institute, National Applied Research Laboratories (Hsinchu, Taiwan). This study was approved by the Institutional Review Board of the Kaohsiung Medical University Hospital (KMUHIRB-SV(I)-20170012). The study protocol is registered in ClinicalTrials.gov (ClinicalTrials.gov Identifier: NCT04407351). The algorithm of the study design is shown in Figure 1.

### 2.2. Measurement of Cutaneous Microcirculation

The cutaneous microcirculation of each finger was measured by LDF. The method for evaluation of skin microcirculation has been described previously [3,16]. Briefly, the LDF (PeriFlux System 5000; Perimed, Stockholm, Sweden) with plastic holder was used for evaluation of cutaneous flow of each finger. The probe with plastic holder was placed on the ventral side of the 1st phalange of each finger. The blood flow was determined by the product of the number of red blood cells moving in the measured volume (within the surface capillaries of the skin) and the mean velocity of these blood cells. This is expressed as perfusion unit (PU), which is arbitrarily determined by the Perimed analysis program of PeriFlux to show real-time dynamic change of PU and allow direct comparison and documentation of blood flow within the experiment [34]. The principles of LDF have been described elsewhere [35,36].

### 2.3. Statistical Analysis

We compared the baseline data among CID, AD, and MCI patients. Sex and comorbid diseases were analyzed with the chi-square test and Fisher’s exact test. Continuous variables including age, body height, body weight, body mass index (BMI), and baseline finger microcirculation were compared by one-way analysis of variance (ANOVA). Paired sample t-tests were utilized to compare change in finger microcirculation before and after light therapy. Because the data contained repeated observations, the dependent variables such as finger microcirculation (laser Doppler perfusion), were measured at 10 fingers 3 times (baseline, after the first light therapy, and the second cross-over light therapy, Figure 1) for each participant; instead of multiple regression, we used the generalized estimating equation (GEE) approach to derive the effects of treatments with adjustment of age, gender, and BMI [37,38]. For each participant i, the 10 finger microcirculation perfusion unit (PU) for the 3-time measurements were presented as:

Y_ijk_ = β_1m_·light therapy + β_2k_·measures + β_3n_·diseases+ β_4_·sex + β_5_·age + comorbidity
with or without interaction terms of light therapy and diseases
j = 1, 2, …, 10 fingers
k = 1, 2, 3 measures (baseline, the first measure, and the second measure)
m: 0, 1, 3, with dummy variables for light therapy (baseline, red, and green light)
n: diseases (primary insomnia, mild cognitive impairment, or Alzheimer’s disease)
sex (male versus female)
comorbidity (yes versus no)

The computer package, SAS 9.4, GENMODE procedure was used, and a two-tailed *p*-value < 0.05 indicated statistical significance. To display the complex fitted GEE model, we used ‘EFFECTPLOT’ statement following the SAS GENMODE procedure. The effect plots displayed the effects of light therapy on finger laser Doppler perfusion along 3-time measurements, while holding other covariates at fixed values (adjustment); this could enable easier interpretation and explanation of the resulting model. 

## 3. Results

### 3.1. Demographic Characteristics of Patients with CID, MCI, and AD

Demographic characteristics of patients with CID, MCI, and AD are shown in Table 1. The numbers of patients with CID, MCI, and AD are 20, 17, and 14, respectively. Sex was not significantly different among the three groups. Patients with AD were older than the other two groups (AD vs. MCI vs. CID = 75.0 ± 4.7 vs. 67.3 ± 4.9 vs. 64.1 ± 4.5 years, *p* < 0.001), which is consistent with that fact that MCI may progress to AD at an older age. There was no difference in body height, body weight, and body mass index among the three groups. As for comorbidities, patients with MCI and AD tended to have more comorbid chronic diseases (*p* < 0.01).

### 3.2. Baseline Finger Microcirculation Differs among Patients with CID, MCI, or AD

Table 2 shows baseline microcirculation of each finger measured by LDF in patients with CID, MCI, or AD. Among the ten fingers, right first to fourth fingers and left first to fourth fingers had significant differences in baseline cutaneous microcirculation among the three disease groups. Patients with CID had highest baseline cutaneous microcirculation, while patients with AD had the lowest baseline cutaneous microcirculation. MCI had baseline microcirculation between CID and AD. 

### 3.3. Red Light Significantly Changes Finger Microcirculation in Patients with CID and AD 

**Figure 2:**
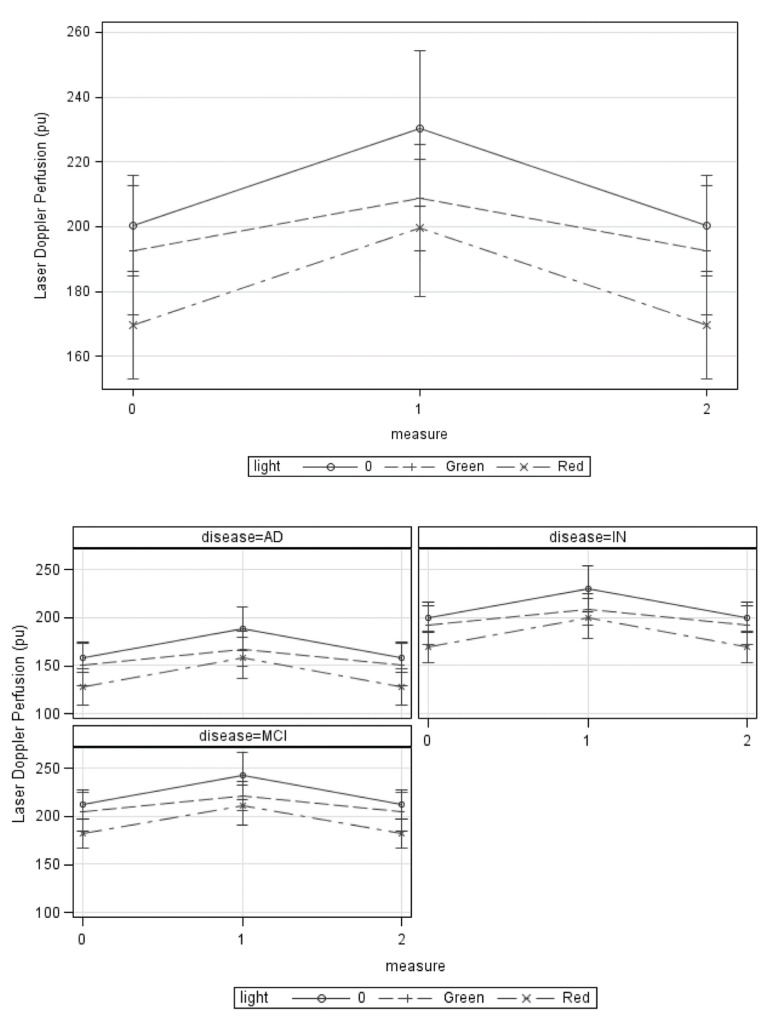
Effect plots of green and red light therapy fitted by generalized estimating equation (GEE) and adjusted for sex, age, and body mass index. The y-axis was laser Doppler perfusion (PU).

Table 3 shows finger perfusion change in patients with CID, MCI, and AD after light therapy. After red light therapy, cutaneous perfusion of fingers in patients with CID significantly decreased (left fingers, before vs. after = 228.99 ± 58.80 vs. 177.41 ± 59.41 PU, *p* = 0.003; right fingers, before vs. after = 227.25 ± 62.00 vs. 162.00 ± 49.34 PU, *p* = 0.007). In patients with AD, red light therapy significantly increased cutaneous perfusion of right fingers (before vs. after = 130.13 ± 49.82 vs. 172.38 ± 38.32 PU, *p* = 0.043). Green light therapy did not significantly change finger perfusion in patients with CID, while patients with AD had higher finger perfusion in left hands after green light therapy (before vs. after = 123.99 ± 58.60 v.s.211.04 ± 68.75 PU *p* = 0.022). Next, we used the GEE method to examine the effects of light therapy with adjustment of age, gender, and BMI. Table 4 shows that red light therapy had significant effects on cutaneous perfusion change (*p* = 0.0042), while the effects of green light did not reach statistical significance (*p* = 0.3012). These findings indicate that red light therapy had significant effects on cutaneous microcirculation, especially in patients with AD compared to CID patients. Effect plots of red light therapy fitted by GEE and adjusted for sex, age, and BMI, show that it had the most effective cutaneous perfusion change. For each group of patients, the same result was found, that red light was the most effective treatment (Figure 2).

## 4. Discussion

The present study showed that baseline cutaneous perfusion differs among patients with AD, MCI, and CID. This difference may reflect changes in sympathetic tone in these diseases. Sympathetic neural activation could induce either vasoconstriction or vasodilatation, depending on the environmental stimuli and upstream hypothalamic control of autonomic nerves [39,40]. During heat exposure, for example, hyperthermia is sensed by the hypothalamus, which subsequently induces cutaneous vasodilation and sweating via sympathetic activation [39,40]. Literature has indicated that CID is accompanied by elevated sympathetic neural outflow as well as reduced parasympathetic tone, which may manifest as augmented neural cardiovascular responsiveness to stress and subsequent cardiovascular diseases [25,41]. We further demonstrated that the autonomic dysfunction in patients with CID may be accompanied by increased perfusion in fingers that could be detected by LDF. On the other hand, patients with AD are associated with reduced sympathetic activation [42,43], and we observed that baseline cutaneous perfusion was lower in patients with AD compared to patients with CID. Our findings that patients with AD, MCI, and CID have different baseline cutaneous microcirculation suggests that sympathetic dysregulation might play an important role in neurodegenerative diseases apart from sleep disorders. Further studies are warranted to investigate the mechanisms underlying how the central nervous system regulates sympathetic innervation in different pathogenic conditions.

Red light therapy significantly changed cutaneous microcirculation in patients with CID and patients with AD. The cutaneous perfusion in patients with CID decreased, while the cutaneous perfusion in patients with AD increased after red light therapy. Although reduced sympathetic activity has been reported in patients with AD [27,42,43], there have been conflicting results in different literature. For example, Aharon-Peretz et al. reported that increased sympathetic and decreased parasympathetic cardiac innervation were observed in patients with AD [44]. The status of the autonomic response in patients with AD may depend on cognitive performance. Specifically, Perpetuini et al. reported stronger sympathetic activity in patients with AD compared to healthy controls during the execution of clinical cognitive and mnemonic tests, while lesser sympathetic activity during the Free and Cued Selective Reminding Test (FCSRT) was observed in patents with AD with respect to healthy controls [43,45]. Taken together, the autonomic status in AD may vary with no clear predominance of sympathetic or parasympathetic activity.

Notably, the cutaneous perfusion after red light therapy in patients with CID was similar to that in patients with AD, suggesting that red light therapy may normalize the dysregulation of sympathetic innervation of cutaneous microcirculation. On the other hand, green light therapy did not significantly change finger perfusion in patients with CID but increased finger perfusion in patients with AD. It is well established that green light exposure in the morning has a significant phase-advanced effect on the circadian rhythm. On the contrary, red light exposure has no effect on the circadian rhythm. Thus, we hypothesized that green light therapy through the eyes activates the cutaneous sympathetic vasodilation system through advancing the phase response curve of the circadian rhythm in the hypothalamus. On the contrary, red light therapy, based on its neutral effect on the circadian rhythm, stimulates the hypothalamus to modulate autonomic response without resetting the biological clock [46]. Further experiments need to be conducted to clarify the underlying mechanisms. 

There are several limitations of this study. First, the sample size was small in this study. Further studies with a larger sample size are warranted to confirm the findings observed in this study. Second, demographic characteristics showed that patients with AD were significantly older than patients in other groups. It might be possible that the differences in microcirculation assessed at baseline partially reflected the age difference. Further investigations with age-matched disease groups are needed to obtain data confirming our findings. Finally, we did not evaluate changes in cognitive function after light therapy in patients with CID, MCI, and AD in our study. Further research evaluating the correlations between cognitive function change and microcirculation change after red light therapy will further validate if cutaneous microcirculation could be an indicator of cognitive function improvement.

## 5. Conclusions

Based on our findings, we concluded that cutaneous perfusion is a useful tool to detect sympathetic dysfunction in central nervous diseases, such as CID and AD. Our results also suggest that red light therapy is a potential therapeutic intervention to reverse impaired sympathetic function in patients with CID or AD. The findings that cutaneous microcirculation reflects sympathetic function with underlying central nervous diseases and that red light therapy normalizes the abnormalities in cutaneous microcirculation highlight finger perfusion detected by LDF as a practical tool to investigate brain-skin interactions.

## Figures and Tables

**Table 1:** Demographic characteristics of patients with insomnia, mild cognitive impairment, or Alzheimer’s disease.

	Insomnia (*n* = 20)	Mild Cognitive Impairment (*n* = 17)	Alzheimer’s Disease (*n* = 14)	*p*-Value
Sex (male; female)	17; 3	11; 6	9; 5	0.28 *
Age (years)	64.1 ± 4.5	67.3 ± 4.9	75.0 ± 4.7	<0.001 **
Body height (cm)	158.8 ± 7.4	158.6 ± 6.4	156.1 ± 6.6	0.487 **
Body weight (kg)	61.8 ± 11.7	60.6 ± 9.8	54.4 ± 6.5	0.092 **
BMI ^a^ (kg/m^2^)	24.37 ± 3.30	24.05 ± 3.37	22.30 ± 2.17	0.141 **
Without chronic disease	8	0	0	<0.01 *
With one chronic disease	7	12	13	
With two and more chronic diseases	5	5	1	

* Fisher’s exact test. ** One-way ANOVA. ^a^: body mass index = body weight (kg)/body height^2^ (m^2^).

**Table 2:** Baseline finger microcirculation (laser Doppler perfusion) of patients with insomnia, mild cognitive impairment, or Alzheimer’s disease.

	Insomnia (*n* = 20)	Mild Cognitive Impairment (*n* = 17)	Alzheimer’s Disease (*n* = 14)	*p*-Value *
Right hand				
1st finger	214.65 ± 84.43	190.77 ± 75.23	100.86 ± 51.94	<0.001
2nd finger	243.55 ± 114.26	195.23 ± 83.39	124.55 ± 66.34	0.003
3rd finger	220.87 ± 66.16	236.81 ± 103.38	143.71 ± 49.15	0.004
4th finger	227.20 ± 80.14	196.20 ± 92.57	149.80 ± 73.34	0.035
5th finger	200.79 ± 63.34	234.92 ± 95.75	181.46 ± 65.60	0.154
Left hand				
1st finger	202.21 ± 94.26	228.35 ± 90.58	116.15 ± 62.45	0.002
2nd finger	273.06 ± 85.02	219.61 ± 77.96	124.11 ± 64.64	<0.001
3rd finger	215.36 ± 73.16	233.77 ± 82.84	133.13 ± 58.61	0.032
4th finger	211.40 ± 65.41	227.84 ± 87.48	160.84 ± 72.93	0.048
5th finger	224.89 ± 78.84	216.10 ± 92.22	176.75 ± 85.00	0.254

* One-way ANOVA.

**Table 3:** Finger microcirculation (laser Doppler perfusion) of patients with insomnia, mild cognitive impairment, or Alzheimer’s disease before and after red light or green light therapy.

	Red				Green			
	***n***	**Before**	**After**	*p*-Value *	***n***	**Before**	**After**	*p*-Value *
Right hand								
CID	12	227.25 ± 62.00	162.00 ± 49.34	0.007	13	219.68 ± 47.52	197.04 ± 64.12	0.322
MCI	13	226.56 ± 57.53	198.16 ± 56.49	0.123	14	228.21 ± 55.62	224.28 ± 48.45	0.700
AD	8	130.13 ± 49.82	172.38 ± 38.32	0.043	7	129.99 ± 53.37	173.80 ± 48.53	0.094
Left hand								
CID	12	228.99 ± 58.80	177.41 ± 59.41	0.003	13	221.41 ± 54.22	200.45 ± 57.74	0.258
MCI	13	232.42 ± 71.64	206.78 ± 70.95	0.285	14	233.07 ± 68.87	219.44 ± 48.79	0.261
AD	8	126.15 ± 57.33	175.49 ± 59.73	0.104	7	123.99 ± 58.60	211.04 ± 68.75	0.022

CID, chronic insomnia disorder. MCI, mild cognitive impairment. AD, Alzheimer’s disease. * Paired-t test.

**Table 4:** Analysis of generalized estimating equation (GEE) for laser Doppler perfusion (PU) changes of patients with insomnia, mild cognitive impairment, or Alzheimer’s disease treated by red light or green light therapy.

Parameter		Estimate	Standard Error	95% Confidence Limits		*p*-Value
Intercept		203.6636	44.5965	116.2560	291.0712	<0.0001
Light	Green	5.0476	4.8822	−4.5213	14.6165	0.3012
	Red	−15.4375	5.3907	−26.0032	−4.8719	0.0042
Disease ^†^	AD vs. CID	−38.2216	9.3793	−56.6047	−19.8386	<0.0001
	MCI vs. CID	11.2667	6.4869	−1.4473	23.9807	0.0824
Sex		7.1848	6.8221	−6.1863	20.5558	0.2923
Age		−0.3284	0.5759	−1.4571	0.8003	0.5685
BMI ^‡^		0.7606	1.0003	−1.2001	2.7212	0.4471

^†^ AD, Alzheimer’s disease; CID, chronic insomnia disorder; MCI, mild cognitive impairment. ^‡^ BMI, body mass index = body weight (kg)/(height, m)^2^.

## Data Availability

The data presented in this study are available on request from the corresponding author. The data are not publicly available due to privacy.
